# Mental Healing

**Published:** 1910-07-30

**Authors:** 


### Mental Healing.

Mental healing in our generation has "come to stay," and it is of the utmost importance that the medical profession should make up its mind quickly as to what attitude it should take up on this matter both for its own sake and also for the sake of the public. No doubt can exist in the minds of the unprejudiced that suggestion, both in the "waking state" and in conjunction with hypnosis, is a powerful remedial agent in the cure of all so-called "functional" disorders, from hysteria to constipation. The drug maniac and the alcoholic have benefited on many occasions by such treatment exercised either through the medium of a powerful personality combined with drugs, or directly by the hypnotist; and other results have been recorded where post-hypnotic suggestion has been the means of curing sleeplessness and bringing relief from pain, even where the cause of the sleeplessness and pain have been definitely organic.

With these results before us it is possible to sympathise with those who are led away by quacks and ill-regulated enthusiasts who claim to cure all diseases by processes which are grouped under various names, such as "Faith Healing," "Mental Treatment," "Psychotherapy," "Christian Science," and "Osteopathy." The success of all such treatments depends on the fact that those who undergo the treatment have faith in its efficacy and in the power of the operator to do what he professes to do. In the case of "Christian Science" the practitioners demand that the patient shall have faith in a supernatural origin for the "power" which flows from them, while in the case of the osteopaths the belief demanded is one in their personal capacity and in their new theory of nerve supply. The osteopaths conceal the mental element in their cure, professing to have no dealings with suggestion, and combined with their mental treatment they give their patients a considerable amount of healthy exercise. But whatever the pretensions of the various sections of people who practise such forms of treatment outside the body of medicine organised, it is apparent that the success of their treatment depends on the receptivity of the patient combined with a capacity for conveying suggestion on the part of the practitioner. It is without doubt that all these people are able to cure certain "functional" disorders and also to bring symptomatic relief to many who are the subjects of grave organic disease. It is the duty of the medical profession as a whole to make up its mind without delay as to what action it must take with regard to treatment by suggestion in all its aspects. The position at present is that a form of treatment as powerful for good or evil as the use of opium and alcohol is permitted to be used by persons quite unskilled in the diagnosis of disease and who are responsible to no one save the administrators of the law, and the law in its present state is curiously inadequate to deal with the situation.

Up to within a very few years ago the attitude of the profession towards suggestion was the typically British one of disbelief in a form of treatment that has existed since the time of the Chaldean priesthood (and probably much longer) simply because it did not believe that occurrences just outside the boundaries of daily experience could be genuine. Now, however, that the attitude is changing, that serious and scientifically minded observers are investigating the phenomena of hypnosis, that the expression "functional" is being applied to fewer and fewer diseases, it is necessary that a form of treatment which may be of service in many morbid states should be controlled entirely by the medical profession, especially as the indiscriminate use of such treatment may be of the greatest possible danger to the community, both from the effect which it may have in cases unsuitable for its application and also from the power which unscrupulous people may obtain over their susceptible fellows who have come under their influence. There may be unscrupulous and immoral persons among the medical profession, but a wholesome fear of the General Medical Council keeps them within reasonable bounds, and their doings are apt to be less mischievous than those of wholly irresponsible and unlicensed practitioners in whose hands the treatment by suggestion in its various forms is permitted to be.

